# 基于微阵列聚焦电泳的糖尿病血样临床阳离子交换高效液相色谱图中血红蛋白A_3_峰位置推测

**DOI:** 10.3724/SP.J.1123.2020.12033

**Published:** 2021-11-08

**Authors:** Zehua GUO, Fang LUO, Si LI, Liuyin FAN, Yixin WU, Chengxi CAO

**Affiliations:** 1.上海交通大学电子信息与电气工程学院仪器科学与工程系, 上海 200240; 1. Department of Instrument Science and Engineering, School of Electronic Information and Electrical Engineering, Shanghai Jiao Tong University, Shanghai 200240, China; 2.上海交通大学生命科学技术学院,微生物代谢国家重点实验室, 上海 200240; 2. State Key Laboratory of Microbial Metabolism, School of Life Science and Biotechnology, Shanghai Jiao Tong University, Shanghai 200240, China; 3.上海交通大学学生创新中心, 上海 200240; 3. Student Innovation Center, Shanghai Jiao Tong University, Shanghai 200240, China; 4.上海交通大学医学院附属瑞金医院内分泌科, 上海 200230; 4. Endocrine Institute, Ruijin Hospital, School of Medicine, Shanghai Jiao Tong University, Shanghai 200230, China

**Keywords:** 阳离子交换高效液相色谱, 等电聚焦, 血红蛋白A_3_, 血红蛋白A_1c_, 血样, 糖尿病, cation exchange high performance liquid chromatography (CX-HPLC), isoelectric focusing (IEF), hemoglobin A_3_ (HbA_3_), hemoglobin A_1c_ (HbA_1c_), blood sample, diabetes

## Abstract

血红蛋白A_1c_(HbA_1c_)是糖尿病诊断的关键生物标志物,目前其常用的分析方法为阳离子交换高效液相色谱法(CX-HPLC, 5/50 mm分离柱),此方法虽然具有稳定、快捷与自动化等众多优点,但临床CX-HPLC(VARIANT Ⅱ system)谱图中仍存在未知峰,尤其干扰HbA_1c_准确测定的谷胱甘肽化血红蛋白A_3_(HbA_3_)在色谱图中的相对位置仍不清楚。针对这一问题,该文以人新鲜血液为样本,首先利用低分辨CX-HPLC对血样进行分析,提示未知峰P3存在。然后通过微阵列等电聚焦(IEF)电泳和高分辨阳离子交换HPLC(Mono-S 5/50 mm分离柱)对血样的主要血红蛋白(Hb)成分进行分析,提示未知峰P3为HbA_3_峰。随后,通过血红蛋白谷胱甘肽化实验,利用HbA_1c_峰降低、HbA_3_峰明显增强这一信息,进一步推测未知峰P3即是HbA_3_峰,其在CX-HPLC中的保留时间在1.50 min左右。最后,结合前期研究讨论了体内应激情况下Hb谷胱甘肽化对HbA_1c_检测的影响。该研究丰富了CX-HPLC的未知峰P3的色谱信息,为更精准诊断糖尿病提供了有价值的参考。

## Abstract (en)

Hemoglobin A_1c_ (HbA_1c_) is a major component of glycated hemoglobin in human red blood cells. It has been proven to be a significant biomarker for the diagnosis of diabetes; its content in fresh red cells in diabetes blood reflects the average level of blood glucose over the previous three months. Thus, HbA_1c_ level has been used for the assessment of long-term glycemic control in diabetes; the level of 6.5% HbA_1c_ has been certified as a critical cut-off for the diabetes diagnosis. The current commonly used method for HbA_1c_ quantification is based on cation-exchange high performance liquid chromatography (CX-HPLC). The method has advantages such as high stability, rapidity, and automation, but there are still some unidentified peaks of Hb species in CX-HPLC (VARIANT Ⅱ system); in particular, the presence of HbA_3_ (a glutathiolated Hb) affects the accurate determination of HbA_1c_. HbA_3_ is usually present in healthy adult blood samples at 2%-4%, but the concentration of HbA_3_ increases due to the protection of erythrocytes from oxidation, resulting in decreased HbA_1c_. However, the relative location of the HbA_3_ peak in the CX-HPLC clinical chromatogram has not been established.

To address this issue, we extracted Hb species from fresh blood samples obtained from a hospital in an anaerobic environment to avoid possible redox reactions of Hb and glutathione. After the extraction, the Hb samples were analyzed using two methods: a low-resolution CX-HPLC (5/50 mm column) currently used for diabetes diagnosis and a high-resolution cationic exchange HPLC (Mono-S 5/50 mm column), to identify the peak corresponding to HbA_3_. The CX-HPLC analysis of fresh blood samples indicated that the unknown peak P3 located between HbA_1c_ and HbA_0_ peaks corresponded to the HbA_3_ peak between HbA_1c_ and HbA_0_ in the Mono-S-HPLC. Microarray isoelectric focusing (IEF) was used for the micro-preparation of HbA_3_, HbA_1c_, and HbA_0_ in healthy blood samples; then, the micro-prepared species of HbA_3_, HbA_1c_, and HbA_0_ were individually identified via Mono-S-HPLC. The results of the CX-HPLC, Mono-S-HPLC, and microarray IEF experiments indicated that the P3 peak might correspond to HbA_3_. To confirm this, glutathiolated Hb samples were synthesized via acetylphenylhydrazine and analyzed using both the Mono-S- and CX-HPLC systems. The results showed that the content of both glutaminated hemoglobin of HbA_3_ in Mono-S-HPLC and P3 in CX-HPLC increased, implying the peak of P3 with the retention time of 1.50 min in CX-HPLC was the peak corresponding to HbA_3_ in Mono-S-HPLC and microarray IEF.

Based on the above experiments and our previous results, the influence of HbA_3_ on both the analysis of HbA_1c_ in blood samples and the diabetes diagnosis needs to be considered and discussed. The study results are significant for the tentative assignment of peak P3 and for offering more information on diabetes diagnosis using CX-HPLC in the clinical setting.

血红蛋白A_1c_(HbA_1c_)是人类红细胞中糖化血红蛋白(Hb)的主要成分,由葡萄糖分子或其他还原性糖分子与血红蛋白A的*β*链之间通过非酶促缩合反应形成^[1,2]^。在糖尿病筛查研究中,一些研究者利用电泳技术发现了HbA_1c_,并证明HbA_1c_是糖尿病诊断的重要标志物^[3,4,5]^。在人血红细胞的120天细胞生长周期中,HbA_1c_含量与人体内血糖浓度正相关,可以用来反映最近2~3个月时间内的血糖控制。因此,HbA_1c_含量常被用于评估糖尿病的长期血糖控制情况^[3,4,5]^。

目前,人血HbA_1c_检测方法有30种以上^[6,7]^,其中,阳离子交换高效液相色谱(CX-HPLC)是主要方法,能够较好地实现对临床血样HbA_1c_的检测。HPLC具有稳定、线性好和自动化等优点,能够满足大量糖尿病临床诊断的需求^[8]^。在临床血样CX-HPLC图谱中,主要有HbA_1a_、HbA_1b_、HbA_1c_和HbA_0_峰^[9,10]^,但在大多数CX-HPLC图谱中,在主峰HbA_0_的左侧始终存在未知峰(见图1)。Little等^[9]^与Rohlfing等^[10]^指出,未知峰P3的出现常会导致CX-HPLC检测可信度的丧失。但是,目前对CX-HPLC中未知峰P3的信息了解非常少。

**图1:**
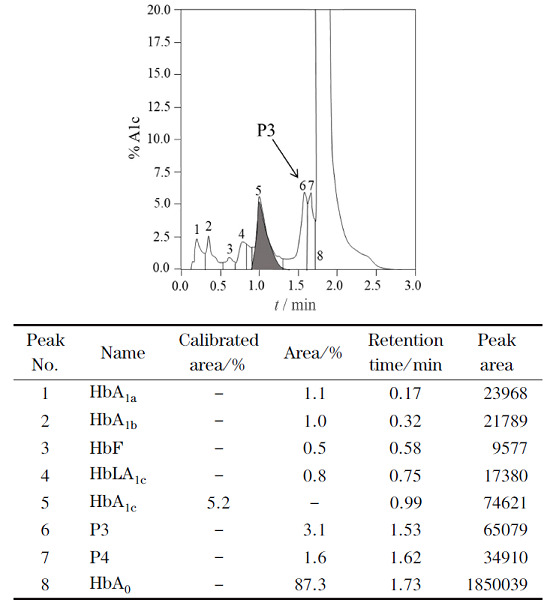
新鲜血样通过VARIANT Ⅱ CX-HPLC系统得到的HbA1c分析结果

研究^[11,12,13]^提示未知峰可能是HbA_3_。HbA_3_是一种谷胱甘肽化的Hb产物,性质较为稳定,在人红细胞中浓度较低^[14,15]^。但在人体遭受强烈的氧化应激时(如吸烟和药物作用),血液中的HbA_1c_会与谷胱甘肽结合,导致HbA_3_浓度显著上升,使HbA_1c_检测结果低于真实值^[16,17,18,19]^,影响临床诊断结果。Jeppsson等^[20]^利用高分辨Mono-S色谱柱实现了HbA_3_与HbA_1c_之间的高效分离。基于微阵列等电聚焦(IEF)电泳,我们^[21,22,23,24]^从真实血样中分离纯化了HbA_3_,并利用质谱和酶联免疫法(enzyme linked immunosorbent assay, ELISA)技术对HbA_3_进行了鉴定和含量测定,结合Mono-S-HPLC技术,证实了HbA_3_的生物合成造成HbA_1c_检测结果的降低^[22]^。虽然HbA_3_在Mono-S-HPLC和微阵列IEF中已得到鉴定,但至今仍不清楚HbA_3_在低分辨CX-HPLC谱图中的相对位置。

针对以上问题,本文在前期工作^[21,22,23,24,25,26]^的基础上结合微阵列IEF和高分辨的Mono-S-HPLC方法进一步推测在临床低分辨CX-HPLC中HbA_3_与P3峰的相关性,讨论了其对糖尿病诊断的影响,为临床研究和潜在应用提供参考。

## 1 实验部分

### 1.1 仪器与试剂

临床Bio-Rad VARIANT Ⅱ全自动血红蛋白分析仪,即现广泛用于临床糖尿病HbA_1c_值检测的CX-HPLC设备(Hercules,美国),该仪器使用阳离子交换柱与Bis-Tris/磷酸盐缓冲液,为全自动封闭型检测设备。Mono-S-HPLC色谱仪为配备了Mono S 5/50 GL阳离子交换色谱柱^[20]^(GE Healthcare Life Sciences,美国)的HPLC系统(Thermo Fisher Scientific,美国)。该仪器为手动开放型检测设备,用户可以对设备、方法和缓冲液进行调整,其中5/50指5/50 mm, 5 mm指色谱柱前的预柱长度,50 mm为该色谱柱长度。Mettler-Toledo Delta 320 pH计(梅特勒-托利多集团,上海)。微阵列IEF设备(上海伯楷安生物科技有限公司,上海),用于红细胞中不同种类的Hb高分辨分离与微量制备纯化,该设备的详细信息详见本课题组其他工作^[22,23,24,25,26]^。

丙二酸二钠(纯度>99%)购自TCI(日本东京)。氯化锂(纯度99%)购自阿法埃莎化学有限公司(天津)。丙烯酰胺(超纯级,纯度>99.9%)购自阿拉丁试剂有限公司(上海)。亚甲基双丙烯酰胺(超纯级,纯度>99.9%)和四甲基乙二胺(TEMED)(纯度>99%)购自Sigma-Aldrich(美国)。IEF微阵列分离柱(pH 6~8,长度20 mm×厚度10 μm×宽度1.2 mm)和载体两性电解质(carrier ampholytes (CA), pH 6~8)购自上海伯楷安生物科技有限公司(上海)。其他化学药品均为分析纯,购自国药控股化学试剂有限公司(上海)。超纯水使用超纯水系统(亚荣,上海)制备。

### 1.2 样品准备

人体血液样本取自上海交通大学医学院附属瑞金医院参加体检的志愿者,其采集和检测结果的告知均符合医院的操作要求以及医疗道德规范标准。新鲜血样的HbA_1c_结果由临床医院使用的VARIANT Ⅱ CX-HPLC系统检测得到。

血样预处理^[20]^:将全血样品采集到含有EDTA抗凝剂的试管中,4 ℃保存,预处理在采集后24 h内完成。将血样离心15 min以除去血浆。将红细胞悬浮于3倍体积的0.15 mol/L磷酸钠缓冲液(pH 7.4, 0.9% (质量分数)NaCl)中洗涤3次,然后加入0.4 mL四氯化碳并混合15 min,最后将混合物离心(约6000 g)10 min,并提取含Hb的上清液。为了防止Hb在Mono-S-HPLC和微阵列IEF仪器中发生自氧化,向混合溶液通入一氧化碳1~2 min使其饱和,4 ℃保存。血样中Hb的谷胱甘肽化参照文献^[22,27]^中的方法。取制备好的血样1 mL、0.15 mol/L磷酸盐-氯化钠缓冲液1 mL、10 μmol/L谷胱甘肽1 mL和纯水3 mL混合并调节pH至7.0;然后将混合后的样品(0.5 mL)转移到装有0.2 mg乙酰基苯肼的Eppendorf管中,并在37 ℃下孵育1 h。通过与乙酰苯肼一起孵育,使谷胱甘肽与血红蛋白反应,生成谷胱甘肽化Hb,即HbA_3_。将孵育的混合物离心10 min以去除血红蛋白颗粒沉淀,然后用CX-HPLC和Mono-S-HPLC分析样本中HbA_3_的含量。

### 1.3 HPLC分析

临床低分辨CX-HPLC系统:放置样品至室温,在高离子强度的Bis-Tris/磷酸盐缓冲液环境下,分离柱中的血红蛋白与柱基质由于离子相互作用而分离,最后用光度计检测分离后的血红蛋白在415 nm波长处的吸光度。

高分辨Mono-S-HPLC系统^[20,22]^:分离柱为Mono S 5/50 GL阳离子交换色谱柱;柱温为25 ℃;流动相A为10 mmol/L的丙二酸钠和0.2 g/L叠氮化钠的混合溶液(pH 5.7),流动相B为0.3 mol/L的氯化锂溶液,使用前经0.45 μm微孔滤膜过滤;流速为2 mL/min。洗脱程序:0~5.5 min, 0.2%B; 5.5~7.5 min, 40%B; 7.5~12 min, 50%B; 12~14 min, 100%B; 14~20 min, 0.2%B。检测波长为415 nm,进样量为0.2 μL。

### 1.4 微阵列IEF及微量Hb的制备纯化

微阵列IEF分离血红蛋白:详细IEF分析方法介绍见文献^[22,24]^。所用聚焦电泳分离柱为IEF微分离柱(pH 6~8,长度20 mm×厚度10 μm×宽度1.2 mm)。将处理好的溶血样本与CA混合,最终样本中CA的质量分数为0.4%,血样稀释900倍。电场程序设置:0~2 min, 40 V; 2~4 min, 80 V; 4~6 min, 120 V; 6~120 min, 600 V。进样量为20 μL。

微量Hb制备纯化:同一样本同时进行12根微阵列柱上样,同步进行微阵列IEF聚焦电泳实验^[22,24]^。在微阵列IEF结束后,取出IEF微阵列柱,在解剖镜下利用解剖刀从12根微阵列柱中分别切下HbA_3_、HbA_1c_以及HbA_0_组分。合并同类Hb组分,放置在离心管中用水溶液提取;之后对提取的Hb开展Mono-S-HPLC分析。

## 2 结果与讨论

### 2.1 CX-HPLC未知峰

图1是临床CX-HPLC系统对新鲜血样(24 h内采集)进行分析的结果。样品中HbA_1c_的值为5.2%。在色谱图中HbA_0_峰面积最大,同时也检测了糖化Hb次要成分,包括HbA_1a_和HbA_1b_,但所报告色谱图结果中未提及HbA_3_;正常生理条件下血液中HbA_3_含量通常在2%~4%之间,高于HbA_1a_和HbA_1b_的含量^[28,29,30]^,显然不应将HbA_3_忽略。

### 2.2 基于IEF和Mono-S-HPLC的HbA_3_鉴定

一些Hb变异体会影响阳离子交换HPLC法测量HbA_1c_的结果^[31,32]^,因此,补充HbA_3_信息能增强色谱图的可信度。在对Hb样品进行人工谷胱甘肽化之前,首先用微阵列IEF对含有5.2% HbA_1c_的血液样品进行分析^[22,23,24]^。如图2所示,根据Battelino等^[32]^的报道和本课题组前期研究结果^[22]^,从阳极侧到阴极侧,得到的主要Hb成分依次为HbA_3_、HbA_1c_和HbA_0_。为了进一步确认,本研究用Mono-S-HPLC对新鲜血样进行再次分析^[20]^,结果如图3a所示,该图显示了HbA_3_存在时血样中各血红蛋白成分的分离情况。此外,利用微阵列IEF技术^[21,22,23,24]^微分离后提取的各个Hb成分再用Mono-S-HPLC进行分析。结果如图3b、3c和3d所示,每个峰保留时间均与混合样品分离得到的峰对应,同时基于前期质谱鉴定和ELISA测定结果^[22]^,图3验证了HbA_3_峰的存在。

**图2:**
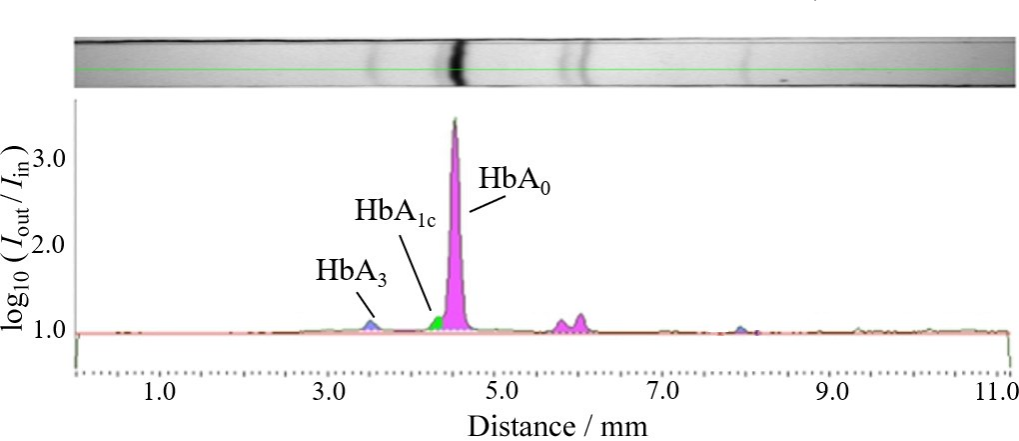
健康成人血红细胞中血红蛋白成分的微阵列IEF电泳图谱

**图3:**
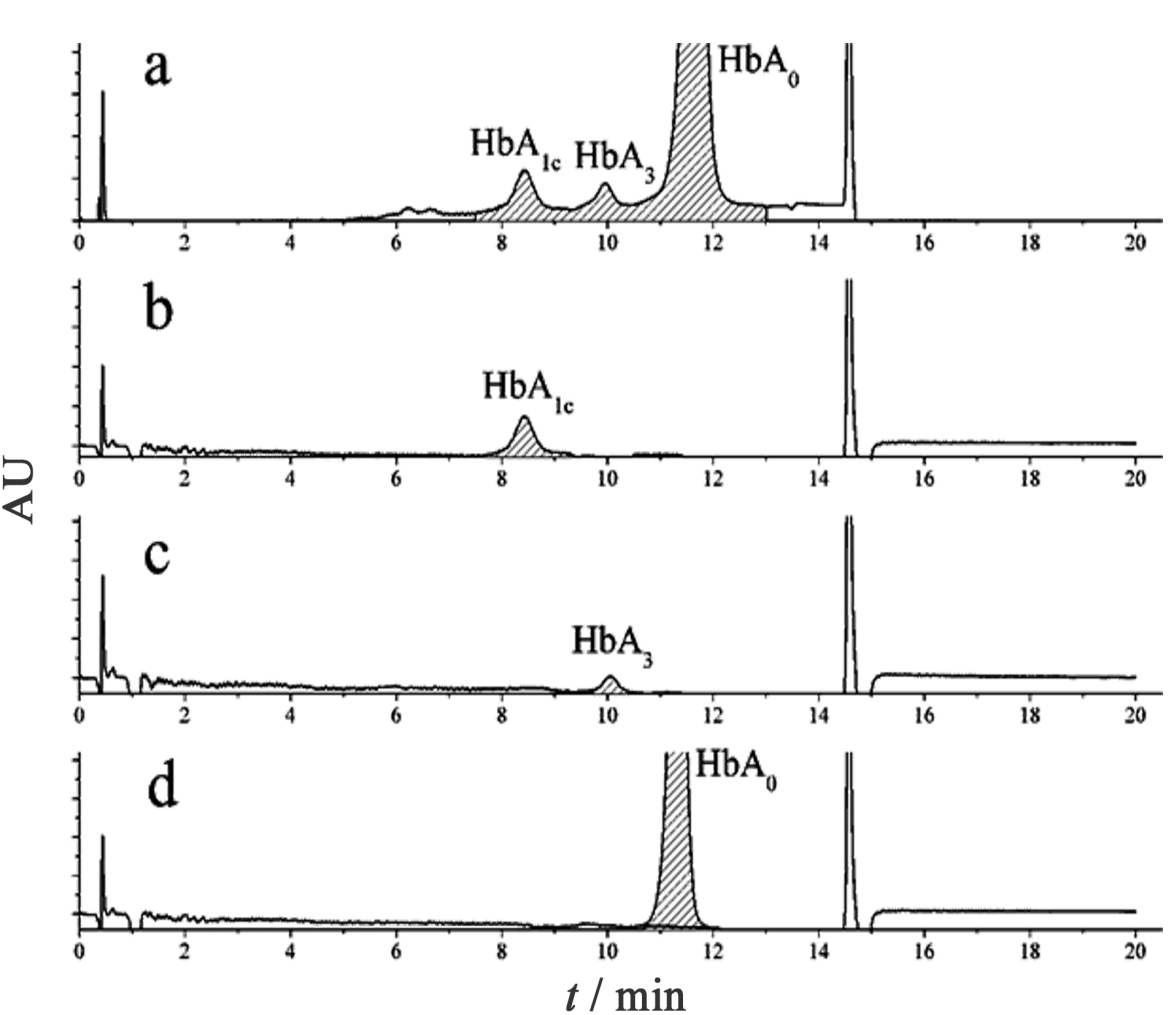
新鲜血样与微阵列IEF分离所得各血红蛋白 成分的Mono-S-HPLC谱图

### 2.3 交叉分析推测P3-HbA_3_峰

为进一步确认CX-HPLC中的P3峰与Mono-S-HPLC的HbA_3_峰的关系,在试管内模拟机体应激过程,加速血液样本中HbA_0_的谷胱甘肽化反应;之后,分别用Mono-S-HPLC和CX-HPLC对谷胱甘肽化的血样进行分析(见图4和图5)。比较图4和图3a可以发现,谷胱甘肽化后HbA_1c_峰明显降低,而HbA_3_峰明显增加;表明谷胱甘肽化降低了血液样品中HbA_1c_的含量,而显著提高了血液样品中HbA_3_含量。图5显示了图1血样谷胱甘肽化后的CX-HPLC结果,与图1相比,图5中大部分峰保留时间基本与图1相对应,但图5中P3峰的峰面积相比图1明显增大,且迁移时间由1.53 min(见图1)延长至1.58 min(见图5),推测图5中的P3峰变化是由于谷胱甘肽化导致的含量增加而使峰的保留时间延后,因而推测P3峰即为HbA_3_峰。同时,P3/HbA_3_相对峰面积明显提高,与Mono-S-HPLC系统得到的结果一致(见图4),再次提示CX-HPLC中的P3峰为Mono-S-HPLC的HbA_3_峰。

**图4:**
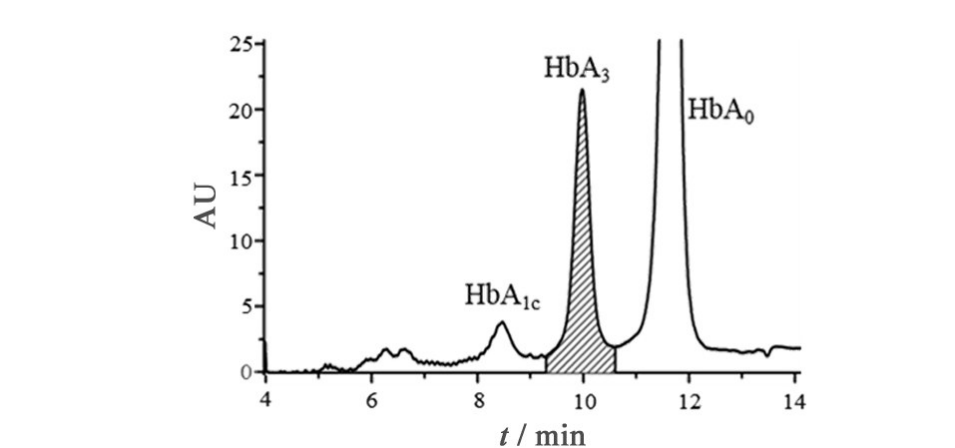
谷胱甘肽化样品的Mono-S-HPLC谱图

**图5:**
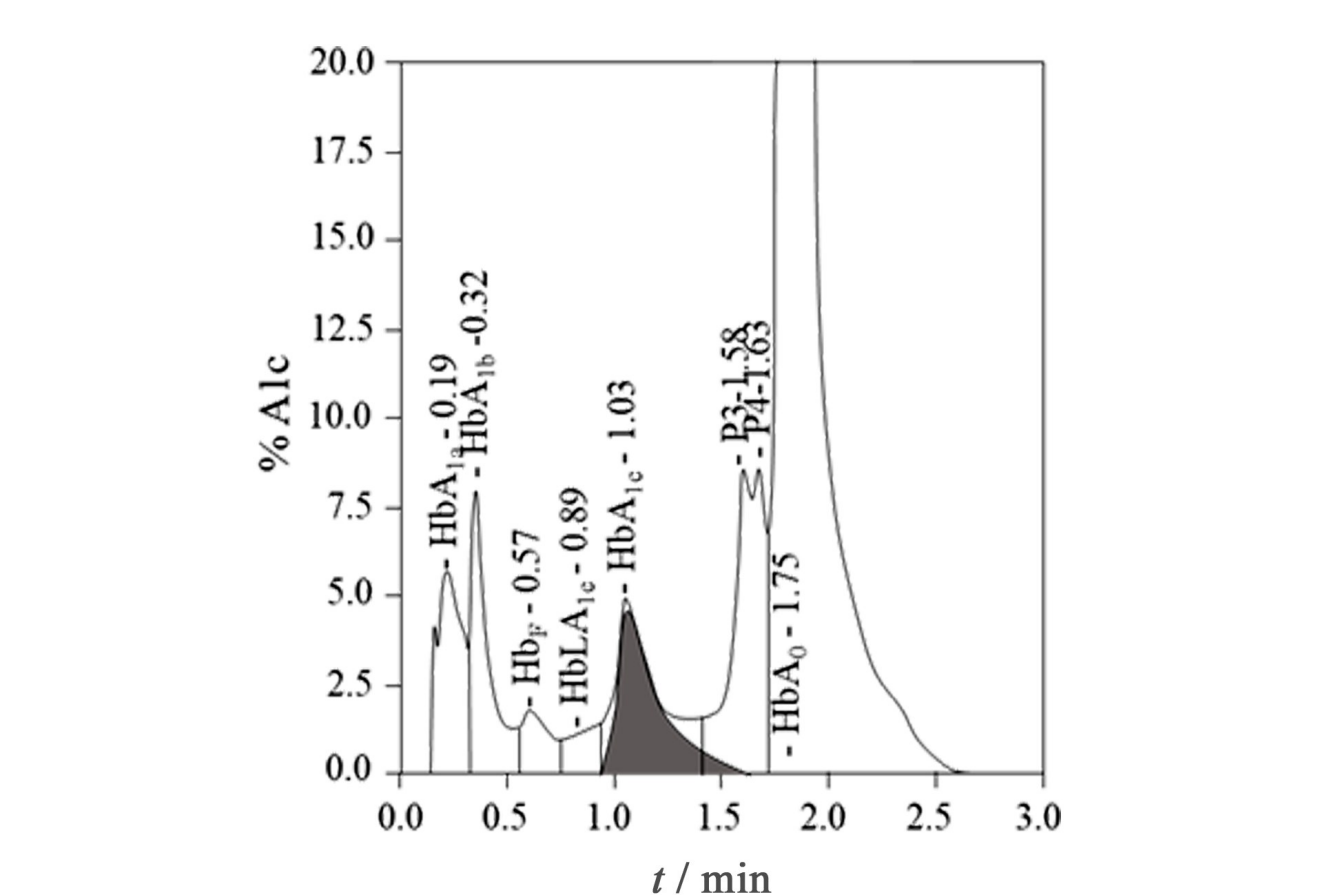
谷胱甘肽化样品通过VARIANT Ⅱ CX-HPLC 系统得到的HbA_1c_分析结果

在本课题组的前期研究中,基于微阵列IEF、质谱技术和ELISA方法,我们证明了血样中HbA_3_会造成HbA_1c_检测结果显著偏低^[22]^,为了提高糖尿病检查中HbA_1c_测定值的准确性,结果中有必要添加HbA_3_峰的信息,包括保留时间和相对峰面积等。但当前在对糖尿病的检测评估中,检验科医师没有考虑P3峰或HbA_3_峰对HbA_1c_峰面积的影响;并且,临床医生依赖自身的经验,不可避免地存在主观因素的影响。本研究推测了HbA_3_峰在CX-HPLC中的相对位置,利用IEF、Mono-S-HPLC推测CX-HPLC中未知P3峰是HbA_3_峰。相关研究结果有望提高糖尿病血样的HbA_1c_检测结果的可信度。

## 3 结论

在本文中,我们通过Mono-S-HPLC、微阵列IEF和临床CX-HPLC系统的交叉分析推测了先前工作^[22]^中提到的未知P3峰即为HbA_3_峰,并确定了其在各个检测系统中的相对位置。此外,本文基于前期工作^[22]^,并通过比较谷胱甘肽化和非谷胱甘肽化血样的交叉分析结果,讨论了HbA_3_对糖基化血红蛋白检测的明显干扰。
